# Supplementary material for: Poly(A) variants supporting robust transmission stability in bacteria and high protein expression in animals for mRNA in vitro transcription
Source: Mol Ther Nucleic Acids. 2025 Dec 22;37(1):102809. doi: 10.1016/j.omtn.2025.102809 (PMC12811438; doi:10.1016/j.omtn.2025.102809)
Supplement: Document S1. Figure S1 and Tables S1–S3 [file mmc1.pdf]

OMTN, Volume *37*

Supplemental information

Poly(A) variants supporting robust transmission stability in bacteria and high protein expression in animals for mRNA *in vitro* transcription

Hua Chen, Wei Qin, Hui Bao, Xuwei Chen, Xu Ye, Yi Wang, Liang Liu, Yanguang Zhang, Ying Sun, Tingting Zhang, Yijie Dong, Shan Cen, and Weiguo Zhang

Supplemental Information

Supplemental Figures with Legends

Figure S1.

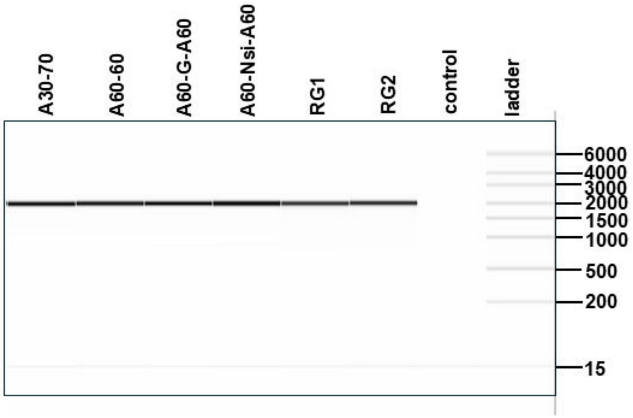

Figure S1. Capillary electrophoresis of synthesized *luciferase* mRNAs with different poly(A) tail variants for LNP preparation and animal study. The names of luciferase mRNAs containing various poly(A) tail variants were indicated on top of corresponding lanes. Four variants from previous work (A30-70, A60-60, A60-G-A60, and A60-Nsi-A60) and two new variants from this study (RG1 and RG2) were included. (*ladder*) The molecular markers of known sizes were used as size control with the sizes shown to the right of the gel image. (*control*) A 15 nt RNA was loaded in the left lane next to ladder, and was included for each lane.

Supplemental Tables

Table S1. Comparison of transmission stability of poly(A) variants 3' to Luc ORF. Transmission stability is calculated as (No. of clones with fewer than five adenosine loss/total No. of clones sequenced) x 100%. The actual number of stable clones and total number of clones sequenced are indicated in parentheses. p-values are calculated between each pair of different poly(A) variants (Chi-square test). "n.s.", not significant; *, $p<0.05$; **, $p<0.01$; ***, $p<0.001$; NA, not applicable. "-" indicates pair-wise comparisons done in the opposite direction in the table.

**poly(A) Variants**	**A30-70**<br>100%<br>(50/50)	**A60-60**<br>95%<br>(95/100)	**A60-G-A60**<br>84%<br>(42/50)	**A60-Nsi-A60**<br>96%<br>(48/50)	**A120**<br>70%<br>(35/50)	**RG1**<br>100%<br>(50/50)	**RG2**<br>100%<br>(62/62)	**RG3**<br>93%<br>(93/100)	**RG4**<br>96%<br>(48/50)	**RG5**<br>92%<br>(46/50)	**RG6**<br>94%<br>(47/50)	**RG7**<br>94%<br>(47/50)	**RG8**<br>94%<br>(47/50)
**A30-70**	NA	-	-	-	-	-	-	-	-	-	-	-	-
**A60-60**	***	NA	-	-	-	-	-	-	-	-	-	-	-
**A60-G-A60**	**	***	NA	-	-	-	-	-	-	-	-	-	-
**A60-Nsi-A60**	ns	***	***	NA	-	-	-	-	-	-	-	-	-
**A120**	***	***	*	***	NA	-	-	-	-	-	-	-	-
**RG1**	ns	***	**	ns	***	NA	-	-	-	-	-	-	-
**RG2**	ns	***	***	*	***	ns	NA	-	-	-	-	-	-
**RG3**	***	ns	***	***	***	***	***	NA	-	-	-	-	-
**RG4**	ns	***	***	ns	***	ns	*	***	NA	-	-	-	-
**RG5**	ns	***	*	ns	***	*	**	***	ns	NA	-	-	-
**RG6**	ns	***	**	ns	***	ns	**	***	ns	ns	NA	-	-
**RG7**	ns	***	**	ns	***	ns	**	***	ns	ns	ns	NA	-
**RG8**	ns	***	**	ns	***	ns	**	***	ns	ns	ns	ns	NA

Table S2. Physical properties of *Luc* mRNA-LNPs with different poly(A) tails used for animal injections and expression. Particle sizes, PDI, encapsulation efficiencies, and *Luc* mRNA content concentrations are provided for each sample.

LNP	Particle size (nm)	PDI	Encapsulation efficiency (%)	mRNA content (μg/mL)
**A30-70**	79.72	0.04694	90.32	147.70
**A60-60**	87.42	0.09351	93.81	111.95
**A60-G-A60**	78.03	0.05652	91.30	104.40
**A60-Nsi-A60**	83.59	0.02673	91.18	109.03
**RG1**	85.69	0.0868	95.22	111.22
**RG2**	88.14	0.05044	97.89	110.47

Table S3. Comparison of transmission stability of poly(A) variants 3' to HPV E6/E7 fusion antigen ORF. Transmission stability is calculated as (No. of clones with fewer than five adenosine loss/total No. of clones sequenced) x 100%. The actual number of stable clones and total number of clones sequenced are indicated in parentheses. p-values are calculated between each pair of different poly(A) variants (Chi-square test). "n.s.", not significant; *, p<0.05; **, p<0.01; ***, p<0.001; NA, not applicable. "-" indicates pair-wise comparisons done in the opposite direction in the table.

**poly(A) Variants**	**A30-70** **98.0% (49/50)**	**A60-60** 69.3% (61/88)	**A60-G-A60** 96.0% (48/50)	**A60-Nsi-A60** 84.0% (42/50)	**RG1** 82.0% (41/50)	**RG2** 98.6% (69/70)	**RG3** 86.1% (87/101)
**A30-70**	NA	-	-	-	-	-	-
**A60-60**	***	NA	-	-	-	-	-
**A60-G-A60**	ns	***	NA	-	-	-	-
**A60-Nsi-A60**	*	***	*	NA	-	-	-
**RG1**	**	***	**	ns	NA	-	-
**RG2**	*	***	**	**	***	NA	-
**RG3**	*	***	***	***	***	***	NA
