# Supplementary material for: Poly(A) variants supporting robust transmission stability in bacteria and high protein expression in animals for mRNA in vitro transcription
Source: Mol Ther Nucleic Acids. 2025 Dec 22;37(1):102809. doi: 10.1016/j.omtn.2025.102809 (PMC12811438; doi:10.1016/j.omtn.2025.102809)
Supplement: Document S2. Article plus supplemental information [file mmc2.pdf]

Molecular Therapy
Nucleic Acids
Original Article

Poly(A) variants supporting robust transmission stability in bacteria and high protein expression in animals for mRNA *in vitro* transcription

Hua Chen,[1,3] Wei Qin,[1,3] Hui Bao,[1,3] Xuwei Chen,[1] Xu Ye,[1] Yi Wang,[1] Liang Liu,[1] Yanguang Zhang,[1] Ying Sun,[1] Tingting Zhang,[1] Yijie Dong,[1] Shan Cen,[2] and Weiguo Zhang[1]

[1]RinuaGene Biotechnology Co., Ltd., Suzhou, Jiangsu, P.R. China; [2]Institute of Medicinal Biotechnology, Chinese Academy of Medical Sciences and Peking Union Medical College, Beijing 100050, China

Poly(A) tail is crucial in regulating mRNA stability and protein translation. Thus, it is an essential element of mRNAs transcribed *in vitro* for mRNA medicines. However, the repetitive nature of the poly(A) tail can lead to significant poly(A) length variation and compromise the quality of the mRNA drug substance. Previous studies improved poly(A) transmission stability by inserting a non-adenosine spacer. Here, we designed new segmented poly(A) variants and evaluated their transmission stability in bacteria transformation and their ability in supporting protein expression in animals. We identified specific variants with multiple non-adenosine insertions that can maintain high transmission stability with robustness in *Escherichia coli* and facilitate high protein expression in animals. Among the newly designed poly(A) variants, RG2 showed high and consistent transmission stability comparable to the A30-70 variant that is the industry gold standard but higher protein expression in animals than A30-70. We also isolate new factors that can influence the stable transmission of poly(A), such as poly(A) surrounding sequences and bacteria culture temperature. Thus, our work offers new tools valuable for rapidly developing mRNA vaccines and therapeutics.

INTRODUCTION

Since the unprecedented COVID-19 pandemic, two mRNA vaccines encapsulated in lipid nanoparticles (LNPs), developed by Moderna and Pfizer/BioNTech, have been administered worldwide, saving millions of lives. Concurrently, numerous other mRNA-based vaccines and therapeutics have been developed for the prevention or treatment of a broad spectrum of diseases, ranging from viral infections to cancer.[1–3] Synthetic linear mRNAs are designed to mimic their eukaryotic counterparts, typically comprising a 5′ cap, 5′ untranslated region (UTR), an open reading frame (ORF), a 3′ UTR, and a long poly(A) tail.[4] The utilization of reliable, universal DNA and RNA *cis*-regulatory elements, such as optimized UTRs and the poly(A) tail, is crucial for the platform features of mRNA therapeutics. This strategy accelerates product development and ensures batch-to-batch reproducibility of *in vitro* transcription (IVT) template DNA and the final mRNA drug substance.

Among the various *cis*-regulatory elements in a linear mRNA molecule, the long homopolymeric poly(A) tail presents a unique challenge. Its highly repetitive nature complicates the procurement of a uniform, high-quality IVT template during plasmid propagation in *Escherichia coli*, which is essential for producing high-quality mRNA drug substances.[5,6] Unpredictable shortening of the poly(A) tract in IVT templates can occur with different ORFs and even between batches of the same plasmid. This variability significantly compromises the consistency of drug quality and the universal applicability of the mRNA technology platform, necessitating stringent monitoring throughout drug development and production.[7]

Another significant hurdle in developing safe and effective mRNA medicines is the moderate level of protein expression, at least partially caused by the intrinsic instability of linear mRNA and the inefficient escape of mRNA-LNPs from endosomes into the cellular cytoplasm.[8,9] Consequently, optimizing the poly(A) tail structure to enhance protein expression in eukaryotic cells is a critical consideration in mRNA design.

The native poly(A) tail is added post-transcriptionally by the poly(A) polymerase in the cell nucleus.[10] It is tightly regulated in eukaryotes and plays a vital role in controlling mRNA stability and protein synthesis.[11] Its length averages approximately 200 nucleotides and is dynamically regulated by a balance between poly(A) polymerase activity and the deadenylation complexes Pan2-Pan3[12] and Ccr4-NOT.[13] When the tail shortens to a critical length of ∼10–12

Received 30 March 2025; accepted 17 December 2025;
https://doi.org/10.1016/j.omtn.2025.102809.

[3]These authors contributed equally

Correspondence: Yijie Dong, RinuaGene Biotechnology Co., Ltd., Suzhou, Jiangsu, P.R. China.
E-mail: yijie.dong@rinuagene.com
Correspondence: Shan Cen, Institute of Medicinal Biotechnology, Chinese Academy of Medical Sciences and Peking Union Medical College, Beijing 100050, China.
E-mail: shancen@imb.pumc.edu.cn
Correspondence: Weiguo Zhang, RinuaGene Biotechnology Co., Ltd., Suzhou, Jiangsu, P.R. China.
E-mail: weiguo.zhang@rinuagene.com

© 2025 RinuaGene Biotechnology Co., Ltd.
Published by Elsevier Inc. on behalf of The American Society of Gene and Cell Therapy.
This is an open access article under the CC BY license (http://creativecommons.org/licenses/by/4.0/).

adenosines, it can trigger the removal of the 5′ cap by decapping enzymes, initiating 5′-to-3′ mRNA degradation.[11,14] The poly(A) tail enhances protein synthesis through several mechanisms.[11] A key mechanism involves the poly(A) binding protein (PABP), which binds to a minimum of 12 consecutive adenosines. PABP subsequently interacts with the translation initiation factor eIF4G, facilitating the formation of a closed-loop mRNA structure that stimulates translation initiation.[15,16] Conventionally, it has been thought that poly(A) tails contain only adenosines. However, recent studies found other nucleotides present at low frequencies in the poly(A) tails of many cellular mRNAs,[17,18] with guanosine as the most frequent non-adenosine nucleotide in both animals and plants.[17,19] Incorporated guanosines may enhance mRNA stability by directly counteracting deadenylase or by promoting LARP (La-related protein) recruitment to assist PABP functions.[20]

For mRNA vaccine development, several methods for adding the poly(A) tail to linear mRNAs have been established, primarily categorized as co-transcriptional and post-transcriptional tailing.[1,21] In co-transcriptional tailing, a poly(A) tract of 70–120 nucleotides is encoded directly within the plasmid DNA template and is transcribed as part of the mRNA by T7 RNA polymerase.[1] In contrast, post-transcriptional tailing involves the enzymatic addition of the poly(A) tract to the 3′ end of pre-synthesized linear mRNA using poly(A) polymerase.[1,21] While this method circumvents the issue of plasmid instability in bacteria, it is prone to non-specific nucleotide additions, leading to low batch-to-batch consistency. It also presents significant challenges for process validation during drug production, alongside high costs and complex manufacturing steps. Alternatively, circular RNA technology, which does not require a poly(A) tail for expression,[22,23] inherently avoids the issue of template instability. However, circular RNAs face considerable hurdles in expression efficiency, production yield, and high cost compared to linear mRNAs. Therefore, co-transcriptional tailing remains the dominant method for generating high-quality mRNA drug substances for vaccines and therapeutics.

To address the issue of template instability, several poly(A) variants have been engineered. These designs incorporate non-adenosine spacers within the poly(A) tract to disrupt the homopolymeric sequence, thereby stabilizing plasmid transmission in *E. coli* while preserving the regulatory functions of the poly(A) tail in eukaryotic cells.[24–26] Studies have demonstrated that the length, sequence composition, and relative position of the non-adenosine spacer are critical factors determining the transmission stability of these variants. For instance, the Pfizer/BioNTech COVID-19 mRNA vaccine employed an A30-70 variant, where a 10-nucleotide non-adenosine spacer is inserted after 30 adenosines, effectively reducing the recombination frequency of the IVT template plasmid.[26] The high stability and successful real-world application of this variant have established it as an industry gold standard. While other poly(A) variants have been reported to confer stable transmission in bacteria and enhance protein expression in cultured cells,[24,25] their robustness, especially their ability to maintain stable transmission when combined with diverse ORFs, remains unclear. This robustness is crucial for the rapid development of broad mRNA medicines. Furthermore, the performance of these variants in supporting protein expression *in vivo* is largely unknown. This is another important consideration, as most cancer cell lines used for *in vitro* studies harbor numerous mutations that are not present in normal healthy animals, which could potentially modulate poly(A) tail function. Therefore, there is a clear need for novel poly(A) variants that support both robust transmission stability in bacteria and high-level protein expression *in vivo*.

In this study, we designed multiple segmented poly(A) variants with the aim of identifying new structures fulfilling these requirements. We investigated factors influencing the transmission stability of these variants, including the number, length, and nucleotide composition of the spacers, the surrounding sequence context, and bacterial culture temperatures. Our approach involved an initial evaluation of primary candidates through a preliminary transformation experiment, followed by rigorous assessment of their transmission stability in biological repeats. The most promising variants were then assessed for their impact on luciferase expression in mice following administration of mRNA-LNPs. Among our new designs, the RG2 poly(A) variant demonstrated high and robust transmission stability comparable to the A30-70 tail, while also conferring superior protein expression *in vivo*. We conclude that these novel poly(A) variants are valuable new tools for the research and development of mRNA therapeutics.

RESULTS

Design of new segmented poly(A) variants with multiple non-adenosine spacers

To identify novel poly(A) variants that not only ensure stable transmission in bacteria but also support high levels of protein expression in animals, we designed eight new segmented poly(A) tail variants featuring multiple non-adenosine spacer insertions (Figure 1). Among them, the RG1 variant was engineered to further reduce the length of consecutive adenosine tracts. It features two single guanosine residues inserted within the 5′ A60 tract, creating a pattern of 19A-G-19A-G-19A, followed by a 6-nt spacer and the 3′ 60A tract. Structurally mirroring RG1, the RG2 variant incorporates the two guanosine residues into the 3′ A60 tract, resulting in a 5′ 60A tract, followed by the spacer and a 3′ tract structured as 19A-G-19A-G-17A. The RG3 variant introduces further complexity by including three single guanosine insertions in the second A60 tract of the RG2 design, in addition to replacing the 6-nt spacer with a single guanosine.

Building upon the RG1 architecture, we generated the RG4 and RG5 variants by substituting the two single guanosines in the 5′ A60 tract with cytidines or thymidines, respectively. To assess the impact of spacer sequence, we created the RG6 and RG7 variants by replacing the original 6-nt spacer in RG1 with two distinct 6-nt sequences. Finally, the RG8 variant was designed to evaluate the effect of spacer length by replacing the 6-nt spacer in RG1 with a 12-nt spacer.

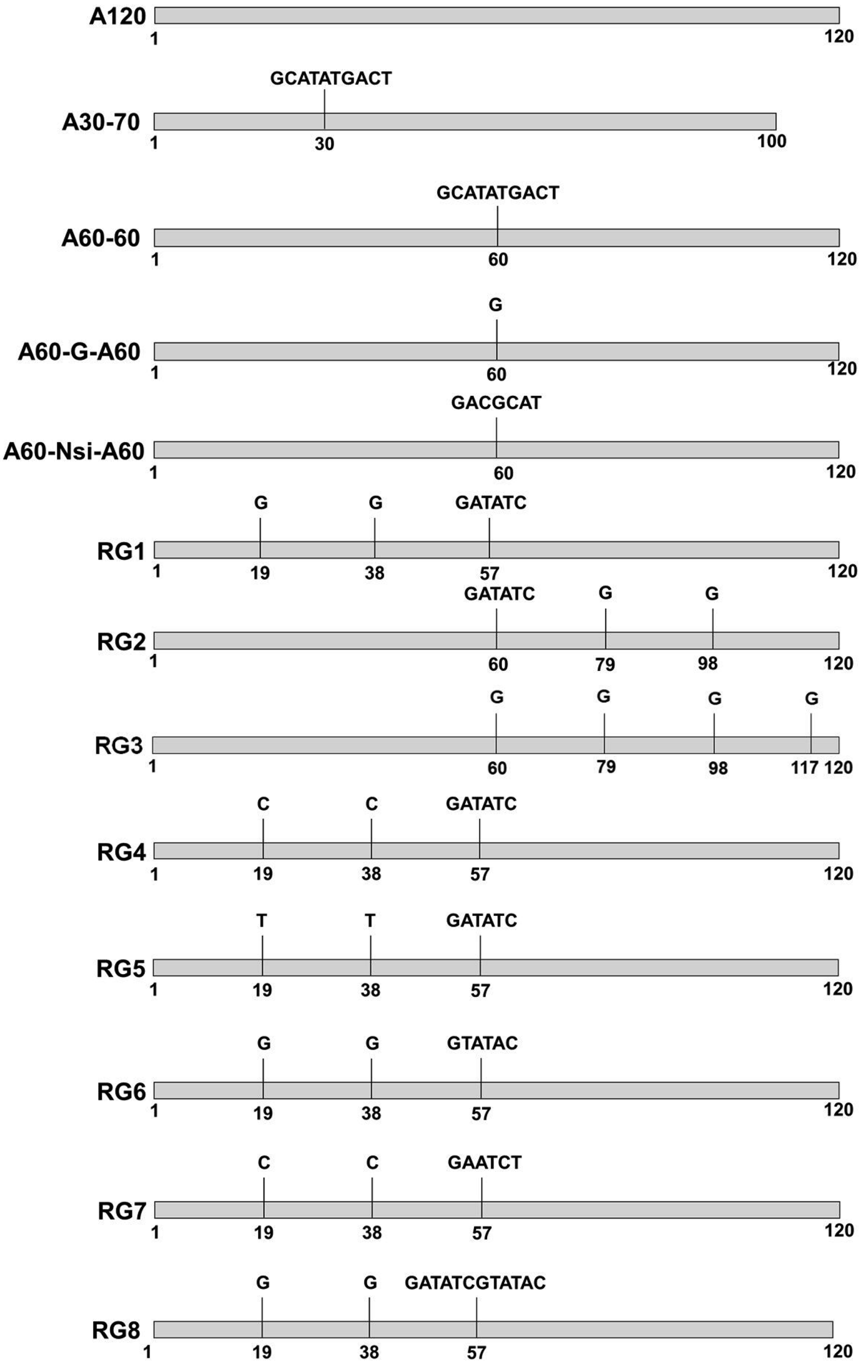

Figure 1. Schematic representation of the poly(A) tail variants used in this study

The names are indicated to the left of the pure 120 adenosine poly(A) tail (A120) or each segmented poly(A) tail variant. For each poly(A) tail variant, the first, the last, and the residues where non-adenosine spacers are inserted are indicated with the location number without counting the non-adenosine insertions. The sequence of each non-adenosine spacer is shown above the insertion site.

For comparative purposes, several previously reported poly(A) variants are illustrated as controls. The variants A60-G-A60, A60-Nsi-A60, and A60-60 contain non-adenosine spacers of 1, 6, and 10 nt, respectively, positioned between two A60 tracts. In contrast, the A30-70 control variant features the same 10 nt spacer as A60-60 but is located between A30 and A70 tracts (Figure 1).[25,26]

Preliminary characterization of new poly(A) tail variants on transmission stability

We first compared the transmission stability of our segmented poly(A) variants against published control variants (A60-G-A60, A60-Nsi-A60, A60-60, and A30-70) in a single-round transformation experiment. Each variant was inserted downstream of an *α-globin* 3′ UTR coupled with a firefly luciferase (*Luc*) ORF. All tested variants, including both newly designed and published controls, demonstrated significantly higher transmission stability than the pure A120 poly(A) tail ($p < 0.05$, χ^2 test for each variant; Table S1). Among the previously established variants, A30-70 (100%, 50/50) exhibited the highest stability. Specifically, A30-70, A60-Nsi-A60 (96.0%, 48/50), and A60-60 (95.0%, 95/100) were all more stable than A60-G-A60 (84.0%, 42/50) ($p < 0.05$, χ^2 tests). All newly designed poly(A) variants showed transmission stability exceeding 90%. Notably, two of our variants, RG1 and RG2, not only were statistically comparable to the top-performing A30-70 control but also achieved a perfect 100% transmission stability (50/50 and 62/62, respectively), matching the performance of A30-70 (Table S1).

Analysis of this preliminary dataset revealed several trends. First, spacers of 6 nt or 10 nt appear to confer superior stability compared to single-nucleotide spacers, underscoring the importance of spacer length. For instance, variants with 6 nt or 10 nt spacers, such as A30-70 and RG2, demonstrated significantly higher stability than A60-G-A60 and RG3, which rely on one or multiple single guanosine insertions ($p < 0.05$ for all comparisons; Table S1). Second, the superior stability of RG3 over A60-G-A60 ($p < 0.001$, χ^2 test), despite differing only in the number of guanosine insertions in the second A60 tract, suggests that further reducing the length of consecutive adenosine tracts enhances transmission stability in bacteria. Third, the specific nucleotide sequence of the spacer appears to exert

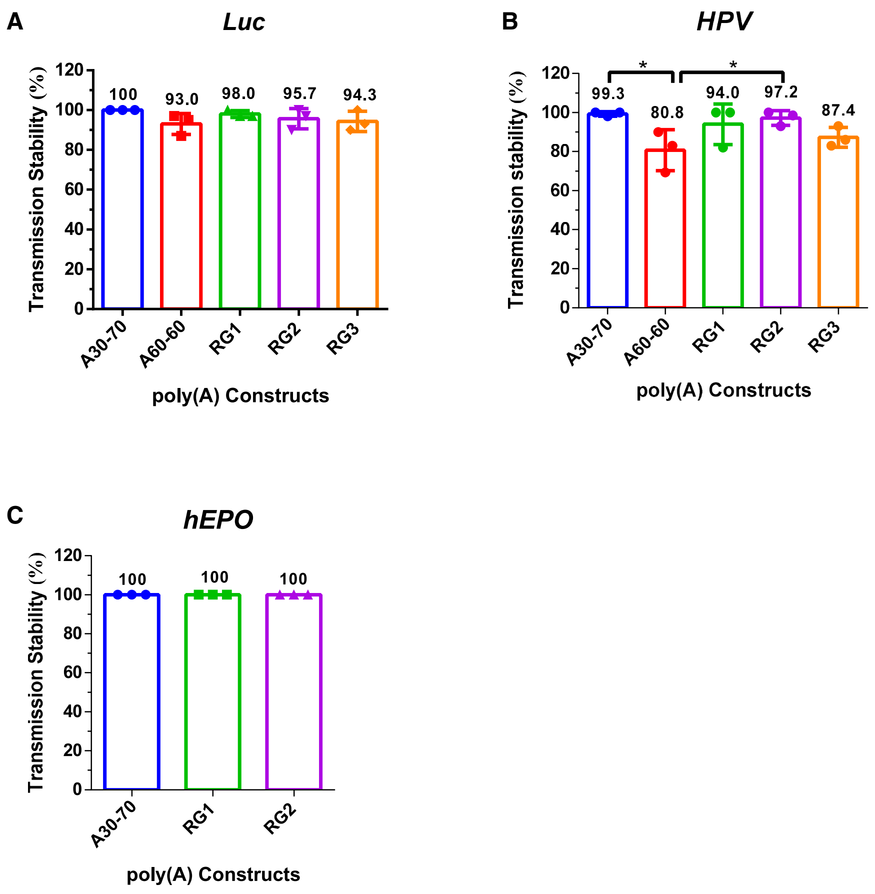

Figure 2. Specific poly(A) variants show high and robust transmission stability in combination with different ORFs

(A) Boxplot showing transmission stability of A30-70, A60-60, RG1, RG2, and RG3 in combination with firefly luciferase (*Luc*) ORF ($n = 3$). (B) Boxplot showing transmission stability of A30-70, A60-60, RG1, RG2, and RG3 in combination with HPV E6/E7 fusion antigen (*HPV*) ORF ($n = 3$). (C) Boxplot showing transmission stability of A30-70, RG1, and RG2 in combination with *hEPO* ORF ($n = 3$). Data are shown as mean ± SEM. *$p < 0.05$, one-way ANOVA. Comparisons without significant differences were not labeled.

a mild influence on stability. Variants RG4–RG7 differ from RG1 only in their single-nucleotide insertion sequences or 6 nt spacer sequences (Figure 1). A similar, though statistically insignificant, trend of slightly lower stability was observed for RG4, RG6, and RG7 relative to RG1, with only the RG5 showing a statistically significant decrease in stability compared to RG1 ($p < 0.05$, χ^2 test). In summary, our preliminary data successfully identified new poly(A) variants with high transmission stability when coupled with the *Luc* ORF, with RG1 and RG2 emerging as the most promising candidates.

Impact of surrounding sequences on the transmission stability of poly(A) variants

The development of a robust mRNA drug platform requires poly(A) tail variants that maintain high transmission stability irrespective of the ORF in use. Therefore, we selected our top candidates, RG1 and RG2, for further characterization in replicate experiments, coupling each with either the *Luc* ORF or an ORF encoding an HPV E6/E7 fusion protein. Several variants, including the published A30-70 and A60-60, as well as the RG3 variant from this study, were included as controls.

When coupled with the *Luc* ORF, the transmission stabilities of the A30-70, A60-60, RG1, RG2, and RG3 variants were 100.0%, 93.0%, 98.0%, 95.7%, and 94.3%, respectively. Under this condition, all tested variants demonstrated comparable transmission stability ($p > 0.05$, one-way ANOVA, $n = 3$) (Figure 2A). A more pronounced effect was observed when the variants were coupled with the HPV fusion antigen ORF. Here, the transmission stabilities were 99.3%, 80.8%, 94.0%, 97.2%, and 87.4% for A30-70, A60-60, RG1, RG2, and RG3, respectively. Notably, RG1 and RG2 remained statistically indistinguishable from A30-70 ($p > 0.05$, oneway ANOVA, $n = 3$) and were significantly more stable than both RG3 and A60-60 ($p < 0.05$, one-way ANOVA, $n = 3$) (Figure 2B). To further assess robustness, we evaluated the top performers A30-70, RG1, and RG2 in combination with a human erythropoietin (*hEPO*) ORF, which is 582 nt in length and has a high GC content of 64%. All three variants exhibited 100% stability across biological triplicates, with no significant differences observed ($p > 0.05$, one-way ANOVA) (Figure 2C). Collectively, these data confirm that A30-70, RG1, and RG2 maintain high transmission stability across diverse ORFs. Furthermore, the results suggest that the stability of certain poly(A) variants, such as A60-60 and RG3, is more susceptible to influence from the upstream ORF sequence.

We also investigated the impact of different 3′ UTRs on transmission stability, using the RG2 variant as a representative example. Comparison of the *HBA1* (*α-globin*), *MALAT1*, and *RPS2* 3′ UTRs across three independent experiments revealed no significant difference in the transmission stability of RG2 (95.7% for *HBA1*, 90.0% for *MALAT1*, and 89.0% for *RPS2*; $p > 0.05$, one-way ANOVA, $n = 3$) (Figure 3). These results indicate that while the ORF can significantly affect the transmission stability of some poly(A) variants, the 3′ UTR appears to exert a comparatively minor influence at least in the case of RG2.

Culture temperature influences the transmission stability of poly(A) variants in bacteria

We next investigated whether bacterial culture temperature affects the transmission stability of selected poly(A) variants. To this end,

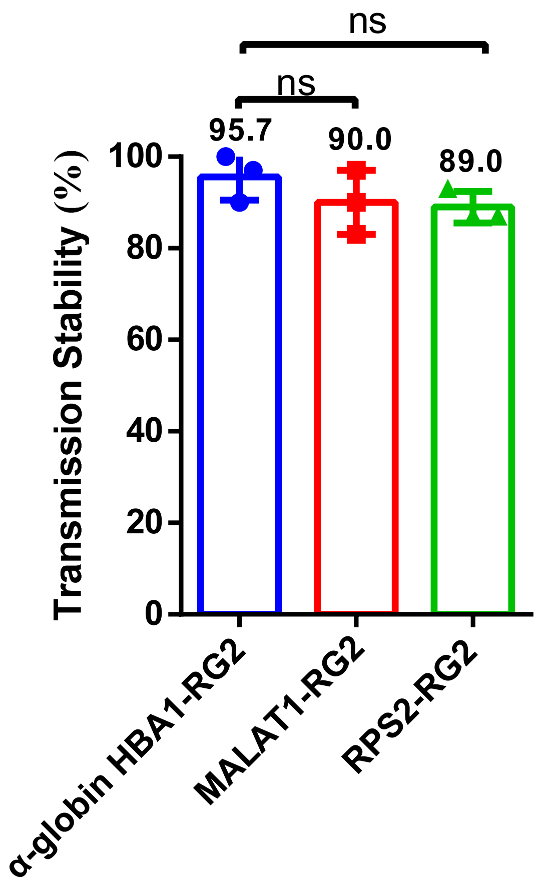

Figure 3. 3′ UTRs affect transmission stability of the RG2 poly(A) variant
Box plot showing transmission stability of RG2 in combination with *α-globin* (*HBA1*), *MALAT1*, or *RPS2* 3′ UTRs ($n = 3$). Data are shown as mean ± SEM. ns, not significant, one-way ANOVA.

we evaluated RG1, RG2, and RG3, alongside the A30-70 and A60-60 controls, across three independent transformation experiments. At a culture temperature of 30°C, all five tested variants exhibited comparable transmission stability ($p > 0.05$, one-way ANOVA). However, when the growth temperature was elevated to 37°C, a significant reduction in transmission stability was observed for the A60-60 variant relative to RG1, RG2, and A30-70 ($p < 0.05$, one-way ANOVA) (Figure 4). This result indicates that elevated growth temperature can preferably compromise the stability of certain poly(A) variants. Notably, the RG1 and RG2 variants demonstrated robust stability at both temperatures, performing comparably to the industry standard A30-70 variant and significantly outperforming A60-60 at 37°C.

Poly(A) variants affect protein expression *in vivo*

Finally, we evaluated the influence of the selected poly(A) variants on luciferase expression *in vivo*. All mRNAs synthesized by *in vitro* transcription (IVT) exhibited integrity exceeding 90% as assessed by capillary electrophoresis, indicating that the various poly(A) tail designs did not interfere with IVT efficiency or mRNA integrity (Figure S1). Lipid nanoparticles (LNPs) encapsulating luciferase mRNA carrying different poly(A) tails, including A30-70, A60-60, A60-G-A60, A60-Nsi-A60, RG1, and RG2, showed comparable encapsulation efficiencies, particle sizes, and polydispersity indices (PDIs) (Table S2).

Each mRNA-LNP formulation was administered intravenously to female BALB/c mice, and whole-body bioluminescence imaging was performed 6 h post-injection. Luciferase mRNA carrying the RG2 tail yielded the highest luminescence signal, which was significantly greater than that of the A30-70 variant ($p < 0.01$, Student's t test) (Figures 5A and 5B). Furthermore, the RG2 variant supported higher luciferase activity than RG1 (1.3-fold increase; $p = 0.08$, Student's t test), as well as relative to the A60-60 and A60-G-A60 variants, though these differences did not reach statistical significance. In contrast, *Luc* mRNA containing the A60-Nsi-A60 variant resulted in the lowest luciferase activity among all tested variants, despite having shown comparable expression to A60-G-A60 in cultured cells in a previous study.[25] Together with the transmission stability data (Figures 2, 3, and 4; Tables S1 and S3), these findings demonstrate that the RG2 variant consistently maintains stable propagation in bacterial systems while also supporting high-level protein expression *in vivo*.

DISCUSSION

In this study, we developed novel segmented poly(A) variants capable of robustly maintaining transmission stability in bacteria while supporting high levels of protein expression in animals. Our approach involved an initial assessment of newly designed variants against published controls in a preliminary transformation experiment to identify promising candidates. The robustness of these leading candidates was subsequently evaluated through repeated experiments under varying conditions, including changes in surrounding sequences and culture temperatures. Finally, we determined the ability of the selected variants to modulate protein expression *in vivo*. These efforts identified several consistently stable novel variants, among which RG2 not only demonstrated bacterial transmission stability comparable to the A30-70 standard but also supported significantly higher luciferase expression in animals. Given that the A30-70 poly(A) variant is regarded as an industry gold standard due to its high transmission stability and efficient protein expression *in vivo*, as evidenced by its application in the BioNTech/Pfizer mRNA-LNP vaccine, our work provides additional valuable tools for the rapid development of mRNA therapeutics.

Robust transmission stability of IVT template DNA is fundamental to leveraging the platform potential of mRNA technology. While several previously reported poly(A) variants exhibited high transmission stability when paired with commonly used reporter ORFs such as luciferase, their ability to maintain this stability under variable conditions remained unclear.[24,25] In this study, the RG1 and RG2 variants consistently demonstrated high stability following ORF or UTR replacements, as well as under elevated culture

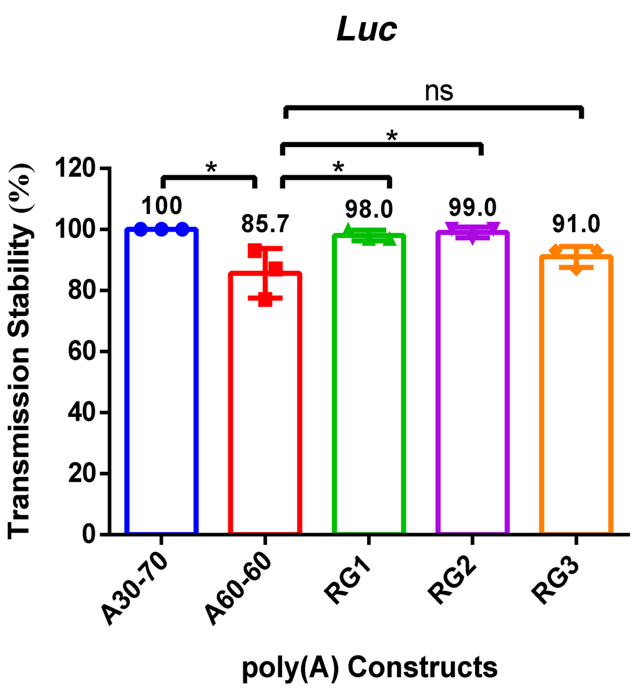

Figure 4. Specific poly(A) variants display robust transmission stability under elevated culture temperatures

Box plot showing transmission stability of A30-70, A60-60, RG1, RG2, and RG3 cultured at 37°C (n = 3). Data are shown as mean ± SEM. $^*p < 0.05$, one-way ANOVA. Comparisons without significant differences were not labeled.

temperatures, performing on par with the A30-70 variant. We believe that these new poly(A) variants hold significant potential to accelerate the research and development of mRNA vaccines and therapeutics.

The development of safe and effective mRNA therapeutics also requires poly(A) variants that can enhance protein expression *in vivo*. Notably, the RG2 variant yielded the highest expression level in mice, significantly surpassing that of A30-70. This finding is particularly important because, among the previously published variants tested here, A30-70 exhibited the highest and most robust transmission stability but only mediocre expression *in vivo* (Figure 5). Other tested variants were either deficient in transmission stability robustness, as observed with A60-60 (Figure 1, Tables S1 and S3), or supported lower protein expression than RG2, as seen with A60-Nsi-A60, A60-G-A60, and RG1, though some differences were not statistically significant, and a potential outlier may have contributed to the reduced expression of the A60-G-A60 variant (Figure 5). Interestingly, while A60-Nsi-A60 was previously reported to support high expression in cell culture,[25] it resulted in the lowest expression in mice among all variants tested here, underscoring the importance of evaluating poly(A) tail function in an *in vivo* context.

The mechanistic basis for RG2's robust transmission stability in bacteria and superior expression *in vivo* compared to A30-70 remains to be fully elucidated. Structurally, RG2 incorporates two single guanosine residues within the latter half of the poly(A) tract, which likely enhances plasmid stability in bacteria by reducing the length of consecutive adenosine repeats. Furthermore, these interspersed guanosines may augment protein expression through multiple potential mechanisms. For instance, the design preserves the minimal tract of 11–12 consecutive adenosines required for PABP binding, thereby promoting translational initiation.[27] Additionally, incorporated guanosines have been shown to counteract deadenylase activity and facilitate the recruitment of La-related proteins (LARPs), which may assist PABP functions.[20] Both mechanisms potentially contribute to the high luciferase expression associated with RG2.

Our work also identified several new factors influencing the transmission stability of poly(A) variants in *E. coli*. Specifically, the surrounding sequences, particularly the ORF, can affect stability in a variant-specific manner, as demonstrated by pairing different poly(A) designs with the *hEPO*, *Luc*, or an HPV antigen ORF (Figure 2). Although the underlying mechanisms require further investigation, we note that these three ORFs possess similar GC contents (58%–64%) but substantially different lengths (0.6 kb for *hEPO*, 1.6 kb for *Luc*, and 2.3 kb for the HPV antigen). Whether ORF length or other sequence features drive the observed differences in transmission stability remains an open question for future research.

In summary, we have identified novel poly(A) variants that combine consistently high transmission stability in bacteria with enhanced protein expression in animals, offering valuable new tools for the rapid development of mRNA vaccines and therapeutics. Future studies aimed at elucidating the underlying mechanisms, coupled with well-designed high-throughput screening strategies, will likely allow more rational design and yield even more effective poly(A) variants.

MATERIALS AND METHODS

Poly(A) variant sequences

Eight new poly(A) variants were designed in this study. Their primary sequences are given below from 5′ to 3′ with non-adenosine spacer sequences underlined:

RG1 (5′-19A-G-19A-G-19A-6 nt spacer-60A-3′)

AAAAAAAAAAAAAAAAAAAGAAAAAAAAAAAAAAAAAAAG
AAAAAAAAAAAAAAAAAAAGATATCAAAAAAAAAAAAAAA
AAAAAAAAAAAAAAAAAAAAAAAAAAAAAAAAAAAAAAAA
AAAAA.

RG2 (5′-60A-6 nt spacer-19A-G-19A-G-17A-3′)

AAAAAAAAAAAAAAAAAAAAAAAAAAAAAAAAAAAAAAAA
AAAAAAAAAAAAAAAAAAAAGATATCAAAAAAAAAAAAAA
AAAAAGAAAAAAAAAAAAAAAAAAAGAAAAAAAAAAAAAA
AAA.

RG3 (5′-60A-G-19A-G-19A-G-19A-G-AAA-3′)

AAAAAAAAAAAAAAAAAAAAAAAAAAAAAAAAAAAAAAAA
AAAAAAAAAAAAAAAAAAAAGAAAAAAAAAAAAAAAAAAA
GAAAAAAAAAAAAAAAAAAAGAAAAAAAAAAAAAAAAAAA
GAAA.

RG4 (5′-19A-C-19A-C-19A-6 nt spacer-60A-3′)

AAAAAAAAAAAAAAAAAAACAAAAAAAAAAAAAAAAAAAC
AAAAAAAAAAAAAAAAAAAGATATCAAAAAAAAAAAAAAA
AAAAAAAAAAAAAAAAAAAAAAAAAAAAAAAAAAAAAAAA
AAAAA.

RG5 (5′-19A-T-19A-T-19A-6 nt spacer-60A-3′)

AAAAAAAAAAAAAAAAAAATAAAAAAAAAAAAAAAAAAAT
AAAAAAAAAAAAAAAAAAAGATATCAAAAAAAAAAAAAAA
AAAAAAAAAAAAAAAAAAAAAAAAAAAAAAAAAAAAAAAA
AAAAA.

RG6 (5′-19A-G-19A-G-19A-6 nt spacer-60A-3′)

AAAAAAAAAAAAAAAAAAAGAAAAAAAAAAAAAAAAAAAG
AAAAAAAAAAAAAAAAAAAGTATACAAAAAAAAAAAAAAA
AAAAAAAAAAAAAAAAAAAAAAAAAAAAAAAAAAAAAAAA
AAAAA.

RG7 (5′-19A-G-19A-G-19A-6 nt spacer-60A-3′)

AAAAAAAAAAAAAAAAAAAGAAAAAAAAAAAAAAAAAAAG
AAAAAAAAAAAAAAAAAAAGAATCTAAAAAAAAAAAAAAA
AAAAAAAAAAAAAAAAAAAAAAAAAAAAAAAAAAAAAAAA
AAAAA.

RG8 (5′-19A-G-19A-G-19A-12 nt spacer-60A-3′)

AAAAAAAAAAAAAAAAAAAGAAAAAAAAAAAAAAAAAAAG
AAAAAAAAAAAAAAAAAAAGATATCGTATACAAAAAAAAA
AAAAAAAAAAAAAAAAAAAAAAAAAAAAAAAAAAAAAAAA
AAAAAAAAAAA.

Four control poly(A) variants based on published studies and patents were included as follows:

A30-70 (5′-30A-10 nt spacer-70A-3′)

AAAAAAAAAAAAAAAAAAAAAAAAAAAAAAGCATATGACT
AAAAAAAAAAAAAAAAAAAAAAAAAAAAAAAAAAAAAAAA
AAAAAAAAAAAAAAAAAAAAAAAAAAAAAA.

A60-60 (5′-60A-10 nt spacer-60A-3′)

AAAAAAAAAAAAAAAAAAAAAAAAAAAAAAAAAAAAAAAA
AAAAAAAAAAAAAAAAAAAAGCATATGACTAAAAAAAAA
AAAAAAAAAAAAAAAAAAAAAAAAAAAAAAAAAAAAAAAA
AAAAAAAAAAAA.

A60-G-A60 (5′-60A-G-60A-3′)

AAAAAAAAAAAAAAAAAAAAAAAAAAAAAAAAAAAAAAAA
AAAAAAAAAAAAAAAAAAAAAGAAAAAAAAAAAAAAAAAA
AAAAAAAAAAAAAAAAAAAAAAAAAAAAAAAAAAAAAAAA
AAAA.

A60-Nsi-A60 (5′-60A-ATGCAT-60A-3′)

AAAAAAAAAAAAAAAAAAAAAAAAAAAAAAAAAAAAAAAA
AAAAAAAAAAAAAAAAAAAAAATGCATAAAAAAAAAAAAA
AAAAAAAAAAAAAAAAAAAAAAAAAAAAAAAAAAAAAAAA
AAAAAAAA.

Gene synthesis and cloning

All poly(A) variants were cloned into a pUC57-based IVT template DNA plasmid (Kan^R). Briefly, the pUC57-Luc construct was synthesized by Genewiz containing pUC57 (GeneBank: Y14837) as the vector backbone and the firefly luciferase ORF (*Luc*) between the *α-globin* gene *HBA1* 5′ and 3′ UTR. Each poly(A) variant was then inserted immediately downstream to the *HBA1* 3′ UTR of the vector by T4 DNA ligase by standard cloning. Briefly, each poly(A) fragment was generated by annealing oligos with short overlaps and with EcoRI and SapI sites at the 5′ and 3′ ends, respectively. The annealing product was then ligated into the pUC57 vector digested by EcoRI (NEB, Cat# R0101S) and SapI (NEB, Cat# R0569S) using T4 DNA ligase (NEB, Cat# M0202S). Positive clones were screened and confirmed by Sanger sequencing at Genewiz.

For ORF cloning, BamHI (NEB, Cat# R0136S) and AscI (NEB, Cat# R0558S) were used to digest the vector and insert fragments, respectively. For 3′ UTR cloning, BamHI and EcoRI sites were used to generate compatible ends between the vector and inserts. T4 DNA ligase was used to ligate the vector and insert DNA digested with the same enzymes. Positive clones were screened and confirmed by Sanger sequencing at Genewiz.

Poly(A) variant transmission stability in *E. coli*

To determine the transmission stability of poly(A) variants, plasmids containing different poly(A) variants were transformed into *E.coli* strain DH5a, and the plates were grown overnight on plates containing kanamycin (50 μg/mL) at 30°C or at 37°C for evaluating the influence of growth temperature. Subsequently, at least 50 colonies were randomly picked for Sanger Sequencing in the preliminary transformation experiment and at least 30 clones for the following biological repeats. Sanger sequencing results were analyzed using the DNAMAN 10 software (Lynnon Biosoft) to determine the numbers of recombined or intact colonies. Considering the actual biological impacts of poly(A) on mRNA expression, functionally stable poly(A) was defined as those missing $\leq$5 adenosines between the 3′ UTR and the 3′ end of the mRNA. The transmission stability of poly(A) was calculated using the following equation: transmission stability (%) = (no. of stable clones/no. of total clones subject to Sanger sequencing) $\times$ 100%.

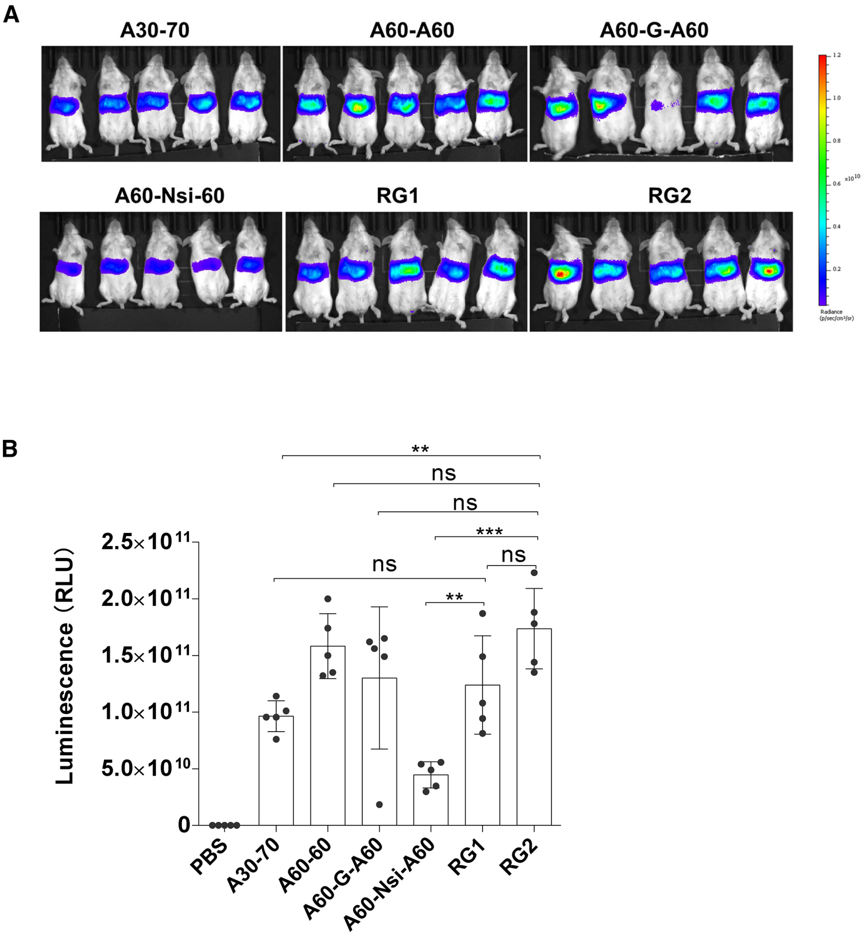

Figure 5. Specific poly(A) variants support high levels of luciferase activity in mice

(A) Whole-body bioluminescence images of mice expressing *Luc* mRNA-LNP with A30-70, A60-60, A60-G-A60, A60-Nsi-A60, RG1, and RG2 poly(A) tails (n = 5 for each), respectively. Mice injected with PBS served as negative controls (n = 5). Images were taken at 6 h after intravenous injection. (B) Quantification of *in vivo* luciferase activity from mRNA-LNP comprising different poly(A) variants using data from (A). Luminescence signals were quantified and presented as mean ± SD. Statistical analysis between A30-70 and each poly(A) variant was performed by Student's t test (**$p < 0.01$; ***$p < 0.001$; ns, not significant).

In vitro transcription

For each IVT reaction, 20 μg of plasmid was linearized with 40 U of BspQ1 (NEB, Cat# R0712L) to generate template DNA ending with poly(A) tail. mRNA was synthesized by IVT using HighYield T7 RNA Synthesis Kit (Cat# ON-40, Hongene Biotech) in a 100 μL reaction mixture for 3 h using 5 μg of linearized plasmid supplemented with 7.5 mM of Cap GAG cap1 analog (Cat# ON-134, Hongene Biotech) and 10 mM of N1-methypseudouridine-5′-triphosphate (Cat# R5-027, Hongene Biotech). mRNA was precipitated using 1/2 volumes of 7.5 M of LiCl at −20°C for 30 min mRNAs were centrifuged at 13,000 rpm for 15 min and resolved in RNase-free H_2O after air-drying.

LNP preparation

LNPs were prepared as previously described.[22] Briefly, the ionizable lipid SM012 (SELLEK, Cat# E044703), cholesterol (SELLEK, Cat# S4154), 1,2-distearoyl-sn-glycero-3-phosphocholine (DSPC) (MCE, Cat# HY-W040193), and DMG-PEG 2000 (Proteintech, Cat# CM03334) were mixed in ethanol at a molar ratio of 50:38.5:10:1.5. The mRNA-LNP was processed by combining the lipid mixture with 20 mM citrate buffer (pH 5.0) containing mRNA (N/P ratio = 6) through a T-mixer (FluidicLab) at total flow rate of 12 mL/min. Particle size and distribution of LNP were measured using a Zetasizer Nano ZS instrument (Malvern). The concentration of mRNA encapsulated into LNPs was analyzed using Quant-iT RiboGreen assay (Cat# R11490, Invitrogen). The efficiency of mRNA encapsulation into LNPs was calculated by comparing measurements in the absence and presence of 1% (v/v) Triton X-100.

Animal experiments

Female BALB/c mice (6–8 weeks, 18–22 g) were purchased from Beijing Vital River Laboratory Animal Technology Co., Ltd. and randomly assigned to the PBS control group (n = 5) or each experimental group (n = 5), followed by tail vein injection with 5 μg of mRNA-LNP for each mouse. D-luciferin substrate was administrated at the dose of 150 mg/kg by intraperitoneally injecting 200 μL of 15 mg/mL D-luciferin substrate (YEASEN, Cat. 40901ES01) at 6 h after injection of mRNA-LNP to assess luciferase expression. Mice were subsequently anesthetized in a chamber supplied with 2.5% isoflurane, then placed on the imaging platform while being maintained on 2% isoflurane via a nose cone. The bioluminescence images were taken 5 min after the D-Luciferin injection using the *in vivo* imaging system IVIS Lumina II (PerkinElmer). The luminescence signal was quantified using Living Image software (v.4.7.4, PerkinElmer).

Animal studies were conducted in accordance with the guidelines of the Chinese Association for Laboratory Animal Sciences and approved by the IACUC of Ascentage Pharma (Approved ethical number: AS-20230621-01, AS-20240313-01, AS-20221228-01).

Statistical analysis

χ^2 tests were performed on the preliminary round of *E. coli* transformation experiment in the Microsoft Office Excel to determine the statistical significance of the differences. One-way ANOVA was performed on biological triplicates of transformation experiments for

transmission stability using GraphPad Prism 10 (GraphPad Software, LLC). Data on the *in vivo* expression of luciferase were analyzed by Student's *t* test using GraphPad Prism 10 (GraphPad Software, LLC).

DATA AND CODE AVAILABILITY

All data are incorporated into the article and its supplemental information.

ACKNOWLEDGMENTS

We thank Ms. Juan Li for LNP preparations, Ms. Na Li for animal injection, and Mr. Qian Dong for legal support. This work was supported by the "Open Competition to Select the Best Candidates" Key Technology Program for Cell Therapy of NTICB (grant no. NCTIB2023XB02010) and CAMS Innovation Fund for Medical Sciences 2021-I2M-1-038 (to S.C.) and 2022-I2M-2-002 (to S.C.).

AUTHOR CONTRIBUTIONS

H.C. and W.Z. designed experiments. H.B., X.C., and Y.S. evaluated transmission stability. W.Q. and X.Y. performed animal experiments. Y.W., L.L., and Y.Z. prepared plasmid DNA. T.Z. prepared all LNPs. H.C. and W.Z analyzed the data and drafted and revised the paper. All authors have read and edited the manuscript.

DECLARATION OF INTERESTS

W.Z., Y.D., H.C., and W.Q. are co-inventors of a patent application describing the poly(A) variants described in this study.

SUPPLEMENTAL INFORMATION

Supplemental information can be found online at https://doi.org/10.1016/j.omtn.2025.102809.

REFERENCES

1. Qin, S., Tang, X., Chen, Y., Chen, K., Fan, N., Xiao, W., Zheng, Q., Li, G., Teng, Y., Wu, M., and Song, X. (2022). mRNA-based therapeutics: powerful and versatile tools to combat diseases. Signal Transduct. Target. Ther. *7*, 166. https://doi.org/10.1038/s41392-022-01007-w.
2. Chaudhary, N., Weissman, D., and Whitehead, K.A. (2021). mRNA vaccines for infectious diseases: principles, delivery and clinical translation. Nat. Rev. Drug Discov. *20*, 817–838. https://doi.org/10.1038/s41573-021-00283-5.
3. Rohner, E., Yang, R., Foo, K.S., Goedel, A., and Chien, K.R. (2022). Unlocking the promise of mRNA therapeutics. Nat. Biotechnol. *40*, 1586–1600. https://doi.org/10.1038/s41587-022-01491-z.
4. Sahin, U., Karikó, K., and Türeci, Ö. (2014). mRNA-based therapeutics–developing a new class of drugs. Nat. Rev. Drug Discov. *13*, 759–780. https://doi.org/10.1038/nrd4278.
5. Preiss, T., Muckenthaler, M., and Hentze, M.W. (1998). Poly(A)-tail-promoted translation in yeast: implications for translational control. RNA *4*, 1321–1331. https://doi.org/10.1017/s1355838298980669.
6. Grier, A.E., Burleigh, S., Sahni, J., Clough, C.A., Cardot, V., Choe, D.C., Krutein, M.C., Rawlings, D.J., Jensen, M.C., Scharenberg, A.M., and Jacoby, K. (2016). pEVL: A Linear Plasmid for Generating mRNA IVT Templates With Extended Encoded Poly(A) Sequences. Mol. Ther. Nucleic Acids *5*, e306. https://doi.org/10.1038/mtna.2016.21.
7. Al Turihi, N., Allouche, D., Quéré, M., Sciuller, M., and Legastelois, I. (2025). Characterization of poly(A) and poly(T) tail lengths in plasmid DNA by liquid chromatography high-resolution mass spectrometry. Anal. Bioanal. Chem. *417*, 59–68. https://doi.org/10.1007/s00216-024-05654-6.
8. Jung, S.J., Seo, J.J., Lee, S., Hyun, S.I., Lee, J.E., Lee, S., Lee, Y., Chang, H., Lee, H., Kim, J.H., and Kim, V.N. (2025). RNA stability enhancers for durable base-modified mRNA therapeutics. Nat. Biotechnol. https://doi.org/10.1038/s41587-025-02891-7.
9. Shi, Y., Mao, J., Wang, S., Ma, S., Luo, L., and You, J. (2025). Pharmaceutical strategies for optimized mRNA expression. Biomaterials *314*, 122853. https://doi.org/10.1016/j.biomaterials.2024.122853.
10. Liu, J., Lu, X., Zhang, S., Yuan, L., and Sun, Y. (2022). Molecular Insights into mRNA Polyadenylation and Deadenylation. Int. J. Mol. Sci. *23*, 10985. https://doi.org/10.3390/ijms231910985.
11. Passmore, L.A., and Coller, J. (2022). Roles of mRNA poly(A) tails in regulation of eukaryotic gene expression. Nat. Rev. Mol. Cell Biol. *23*, 93–106. https://doi.org/10.1038/s41580-021-00417-y.
12. Wolf, J., Valkov, E., Allen, M.D., Meineke, B., Gordiyenko, Y., McLaughlin, S.H., Olsen, T.M., Robinson, C.V., Bycroft, M., Stewart, M., and Passmore, L.A. (2014). Structural basis for Pan3 binding to Pan2 and its function in mRNA recruitment and deadenylation. EMBO J. *33*, 1514–1526. https://doi.org/10.15252/embj.201488373.
13. Collart, M.A. (2016). The Ccr4-Not complex is a key regulator of eukaryotic gene expression. Wiley Interdiscip. Rev. RNA *7*, 438–454. https://doi.org/10.1002/wrna.1332.
14. Garneau, N.L., Wilusz, J., and Wilusz, C.J. (2007). The highways and byways of mRNA decay. Nat. Rev. Mol. Cell Biol. *8*, 113–126. https://doi.org/10.1038/nrm2104.
15. Jackson, R.J., Hellen, C.U.T., and Pestova, T.V. (2010). The mechanism of eukaryotic translation initiation and principles of its regulation. Nat. Rev. Mol. Cell Biol. *11*, 113–127. https://doi.org/10.1038/nrm2838.
16. Gray, N.K., Coller, J.M., Dickson, K.S., and Wickens, M. (2000). Multiple portions of poly(A)-binding protein stimulate translation in vivo. EMBO J. *19*, 4723–4733. https://doi.org/10.1093/emboj/19.17.4723.
17. Lim, J., Kim, D., Lee, Y.S., Ha, M., Lee, M., Yeo, J., Chang, H., Song, J., Ahn, K., and Kim, V.N. (2018). Mixed tailing by TENT4A and TENT4B shields mRNA from rapid deadenylation. Science *361*, 701–704. https://doi.org/10.1126/science.aam5794.
18. Liu, Y., Nie, H., Liu, H., and Lu, F. (2019). Poly(A) inclusive RNA isoform sequencing (PAIso-seq) reveals wide-spread non-adenosine residues within RNA poly(A) tails. Nat. Commun. *10*, 5292. https://doi.org/10.1038/s41467-019-13228-9.
19. Zhao, T., Huan, Q., Sun, J., Liu, C., Hou, X., Yu, X., Silverman, I.M., Zhang, Y., Gregory, B.D., Liu, C.M., et al. (2019). Impact of poly(A)-tail G-content on Arabidopsis PAB binding and their role in enhancing translational efficiency. Genome Biol. *20*, 189.
20. Kozlov, G., Jiang, J., Rutherford, T., Noronha, A.M., Wilds, C.J., and Gehring, K. (2024). Enhanced binding of guanylated poly(A) RNA by the LaM domain of LARP1. RNA Biol. *21*, 764–773.
21. Gote, V., Bolla, P.K., Kommineni, N., Butreddy, A., Nukala, P.K., Palakurthi, S.S., and Khan, W. (2023). A Comprehensive Review of mRNA Vaccines. Int. J. Mol. Sci. *24*, 2700. https://doi.org/10.3390/ijms24032700.
22. Niu, D., Wu, Y., and Lian, J. (2023). Circular RNA vaccine in disease prevention and treatment. Signal Transduct. Target. Ther. *8*, 341. https://doi.org/10.1038/s41392-023-01561-x.
23. Chen, R., Wang, S.K., Belk, J.A., Amaya, L., Li, Z., Cardenas, A., Abe, B.T., Chen, C.K., Wender, P.A., and Chang, H.Y. (2023). Engineering circular RNA for enhanced protein production. Nat. Biotechnol. *41*, 262–272. https://doi.org/10.1038/s41587-022-01393-0.
24. Li, C.Y., Liang, Z., Hu, Y., Zhang, H., Setiasabda, K.D., Li, J., Ma, S., Xia, X., and Kuang, Y. (2022). Cytidine-containing tails robustly enhance and prolong protein production of synthetic mRNA in cell and in vivo. Mol. Ther. Nucleic Acids *30*, 300–310. https://doi.org/10.1016/j.omtn.2022.10.003.
25. Trepotec, Z., Geiger, J., Plank, C., Aneja, M.K., and Rudolph, C. (2019). Segmented poly(A) tails significantly reduce recombination of plasmid DNA without affecting mRNA translation efficiency or half-life. RNA *25*, 507–518. https://doi.org/10.1261/rna.069286.118.
26. Eberle, F., Sahin, U., Kuhn, A., Vallazza, B., and Diken, M. (2020). Stabllization Of poly(A) Sequence Encoding DNA Sequences (Google Patents).
27. Sachs, A.B., Davis, R.W., and Kornberg, R.D. (1987). A single domain of yeast poly(A)-binding protein is necessary and sufficient for RNA binding and cell viability. Mol. Cell Biol. *7*, 3268–3276.

OMTN, Volume *37*

Supplemental information

Poly(A) variants supporting robust transmission stability in bacteria and high protein expression in animals for mRNA *in vitro* transcription

Hua Chen, Wei Qin, Hui Bao, Xuwei Chen, Xu Ye, Yi Wang, Liang Liu, Yanguang Zhang, Ying Sun, Tingting Zhang, Yijie Dong, Shan Cen, and Weiguo Zhang

Supplemental Information

Supplemental Figures with Legends

Figure S1.

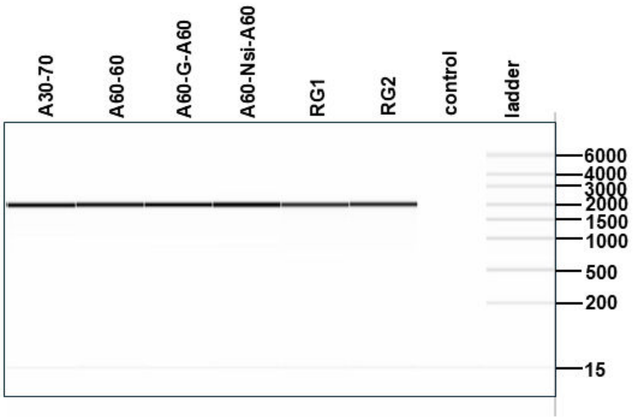

Figure S1. Capillary electrophoresis of synthesized *luciferase* mRNAs with different poly(A) tail variants for LNP preparation and animal study. The names of luciferase mRNAs containing various poly(A) tail variants were indicated on top of corresponding lanes. Four variants from previous work (A30-70, A60-60, A60-G-A60, and A60-Nsi-A60) and two new variants from this study (RG1 and RG2) were included. (*ladder*) The molecular markers of known sizes were used as size control with the sizes shown to the right of the gel image. (*control*) A 15 nt RNA was loaded in the left lane next to ladder, and was included for each lane.

Supplemental Tables

Table S1. Comparison of transmission stability of poly(A) variants 3' to Luc ORF. Transmission stability is calculated as (No. of clones with fewer than five adenosine loss/total No. of clones sequenced) x 100%. The actual number of stable clones and total number of clones sequenced are indicated in parentheses. p-values are calculated between each pair of different poly(A) variants (Chi-square test). "n.s.", not significant; *, p<0.05; **, p<0.01; ***, p<0.001; NA, not applicable. "-" indicates pair-wise comparisons done in the opposite direction in the table.

**poly(A) Variants**	**A30-70** 100% (50/50)	**A60-60** 95% (95/100)	**A60-G-A60** 84% (42/50)	**A60-Nsi-A60** 96% (48/50)	**A120** 70% (35/50)	**RG1** 100% (50/50)	**RG2** 100% (62/62)	**RG3** 93% (93/100)	**RG4** 96% (48/50)	**RG5** 92% (46/50)	**RG6** 94% (47/50)	**RG7** 94% (47/50)	**RG8** 94% (47/50)
**A30-70**	NA	-	-	-	-	-	-	-	-	-	-	-	-
**A60-60**	***	NA	-	-	-	-	-	-	-	-	-	-	-
**A60-G-A60**	**	***	NA	-	-	-	-	-	-	-	-	-	-
**A60-Nsi-A60**	ns	***	***	NA	-	-	-	-	-	-	-	-	-
**A120**	***	***	*	***	NA	-	-	-	-	-	-	-	-
**RG1**	ns	***	**	ns	***	NA	-	-	-	-	-	-	-
**RG2**	ns	***	***	*	***	ns	NA	-	-	-	-	-	-
**RG3**	***	ns	***	***	***	***	***	NA	-	-	-	-	-
**RG4**	ns	***	***	ns	***	ns	*	***	NA	-	-	-	-
**RG5**	ns	***	*	ns	***	*	**	***	ns	NA	-	-	-
**RG6**	ns	***	**	ns	***	ns	**	***	ns	ns	NA	-	-
**RG7**	ns	***	**	ns	***	ns	**	***	ns	ns	ns	NA	-
**RG8**	ns	***	**	ns	***	ns	**	***	ns	ns	ns	ns	NA

Table S2. Physical properties of *Luc* mRNA-LNPs with different poly(A) tails used for animal injections and expression. Particle sizes, PDI, encapsulation efficiencies, and *Luc* mRNA content concentrations are provided for each sample.

LNP	Particle size (nm)	PDI	Encapsulation efficiency (%)	mRNA content (μg/mL)
**A30-70**	79.72	0.04694	90.32	147.70
**A60-60**	87.42	0.09351	93.81	111.95
**A60-G-A60**	78.03	0.05652	91.30	104.40
**A60-Nsi-A60**	83.59	0.02673	91.18	109.03
**RG1**	85.69	0.0868	95.22	111.22
**RG2**	88.14	0.05044	97.89	110.47

Table S3. Comparison of transmission stability of poly(A) variants 3' to HPV E6/E7 fusion antigen ORF. Transmission stability is calculated as (No. of clones with fewer than five adenosine loss/total No. of clones sequenced) x 100%. The actual number of stable clones and total number of clones sequenced are indicated in parentheses. p-values are calculated between each pair of different poly(A) variants (Chi-square test). "n.s.", not significant; *, p<0.05; **, p<0.01; ***, p<0.001; NA, not applicable. "-" indicates pair-wise comparisons done in the opposite direction in the table.

poly(A) Variants	A30-70 98.0% (49/50)	A60-60 69.3% (61/88)	A60-G-A60 96.0% (48/50)	A60-Nsi-A60 84.0% (42/50)	RG1 82.0% (41/50)	RG2 98.6% (69/70)	RG3 86.1% (87/101)
**A30-70**	NA	-	-	-	-	-	-
**A60-60**	***	NA	-	-	-	-	-
**A60-G-A60**	ns	***	NA	-	-	-	-
**A60-Nsi-A60**	*	***	*	NA	-	-	-
**RG1**	**	***	**	ns	NA	-	-
**RG2**	*	***	**	**	***	NA	-
**RG3**	*	***	***	***	***	***	NA
